# Folic acid-conjugated silica capped gold nanoclusters for targeted fluorescence/X-ray computed tomography imaging

**DOI:** 10.1186/1477-3155-11-17

**Published:** 2013-05-29

**Authors:** Zhijun Zhou, Chunlei Zhang, Qirong Qian, Jiebing Ma, Peng Huang, Xi zhang, Liyuan Pan, Guo Gao, Hualin Fu, Shen Fu, Hua Song, Xiao Zhi, Jian Ni, Daxiang Cui

**Affiliations:** 1 Institue of Nano Biomedicine and Engineering, Key Laboratory for Thin Film and Microfabrication of Ministry of Education, Research Institute of Micro-Nano Science and Technology, Bio-X Institutes, Shanghai Jiao Tong University, 800 Dongchuan Road, Shanghai 200240, P. R. China; 2 Department of Surgery, Changzhen Hospital, Second Military Medical University, 155 Fengyang Road, Shanghai 20001, P. R. China; 3 Department of Radiation Oncology, 6th Hospital of Shanghai Jiao Tong University, 600Yishan Road, Shanghai 200240, P. R. China

**Keywords:** Gold nanoclusters, Silica, Folic acid, Fluorescent imaging, X-ray computed tomography

## Abstract

**Background:**

Gastric cancer is 2th most common cancer in China, and is still the second most common cause of cancer-related death in the world. Successful development of safe and effective nanoprobes for in vivo gastric cancer targeting imaging is a big challenge. This study is aimed to develop folic acid (FA)-conjugated silica coated gold nanoclusters (AuNCs) for targeted dual-modal fluorescent and X-ray computed tomography imaging (CT) of in vivo gastric cancer cells.

**Method:**

AuNCs were prepared, silica was coated on the surface of AuNCs, then folic acid was covalently anchored on the surface of AuNCs, resultant FA-conjugated AuNCs@SiO_2_ nanoprobes were investigated their cytotoxicity by MTT method, and their targeted ability to FR(+) MGC803 cells and FR(−) GES-1 cells. Nude mice model loaded with MGC803 cells were prepared, prepared nanoprobes were injected into nude mice via tail vein, and then were imaged by fluorescent and X-ray computed tomography (CT) imaging.

**Results:**

FA-conjugated AuNCs@SiO_2_ nanoprobes exhibited good biocompatibility, and could target actively the FR(+) MGC-803 cells and in vivo gastric cancer tissues with 5 mm in diameter in nude mice models, exhibited excellent red emitting fluorescence imaging and CT imaging.

**Conclusion:**

The high-performance FA-conjugated AuNCs@SiO_2_ nanoprobes can target in vivo gastric cancer cells, can be used for fluorescent and CT dual-mode imaging, and may own great potential in applications such as targeted dual-mode imaging of in vivo early gastric cancer and other tumors with FR positive expression in near future.

## Background

Stomach carcinoma is currently the fourteenth most common cancer in the United States and the second most common cancer in China [1,2], and it is still the second most common cause of cancer-related death in the world [3,4]. Gastric cancer is often either asymptomatic or may cause only no nonspecific symptoms in its early stage. By the times symptoms occur, the cancer has often reached an advanced stage and may have also metastasized, which is one of the main reasons for its relatively poor prognosis. Thus, early recognition and tracking of gastric cancer cells in vivo would be of particular significance [5]. In order to recognize early gastric cancer cells, we tried to select potential biomarkers associated with gastric cancer, and combine nanoparticles and molecular imaging techniques to identify early gastric cancer cells in vivo [6-15]. We also used fluorescent magnetic nanoparticles labeled marrow mesenchymal stem cells and embryonic stem cells to target recognize in vivo gastric cancer cells [7,16-23]. Although some advances have been made, there still existed some unsolvable problems, for example, nanoprobes’ safety, in vivo distribution, metabolism, targeted tumor efficiency, etc., therefore, development of safe and effective nanoprobes for targeted imaging of in vivo gastric cancer has become our major concern.

Presently, a novel noble gold nanoclusters has gained broad attention due to its numerous advantages. Gold nanoclusters (AuNCs) contains tens of atoms with subnanometer dimensions, possess superb red emitting fluorescence property which can avoid in vivo autofluorescent background, and own very low cytotoxicity [24-27]. Thus, AuNCs may be ideal multifunctional imaging contrast reagents. Although AuNCs own obvious advantages, in the course of AuNCs preparation, the excess stabilized proteins may cause the AuNCs solution aggregated together, and resulting in nonspecific absorption. Compared with AuNCs, AuNCs@SiO_2_ is a composite composed of AuNCs core doped in a silica shell, which could protect the AuNCs from aggregation, and make AuNCs easily surface functionalization with different kinds of biomolecules [28-30]. Therefore, it is very necessary to modify AuNCs with silica group as imaging contrast reagent.

Among all the imaging techniques, X-ray computed tomography (CT) is one of the most useful diagnostic tools in hospitals today in terms of availability, efficiency, and cost [31]. CT is able to identify anatomical patterns and to provide complementary anatomical information including tumor location, size, and metastasis based on endogenous contrast [32]. Based on CT technology, functional CT can provide both anatomic and functional information with the appropriate X-ray contrast agent [33]. However, present CT contrast agents are predominantly based on iodine (Z = 53) containing molecules, which have very short blood half-live (<10 min) and moderate atomic number which limit their ability to produce CT contrast signals detected by current CT technology. Moreover, they nonspecifically target tumor tissues because they are not conjugated with most biomarkers and they allow only very short imaging times due to rapid clearance by the kidney. Therefore, the development of tumor targeting X-ray contrast agents is very necessary. So far no report is closely associated with use of FA-conjugated AuNCs as CT contrast reagent.

**Figure 1:**
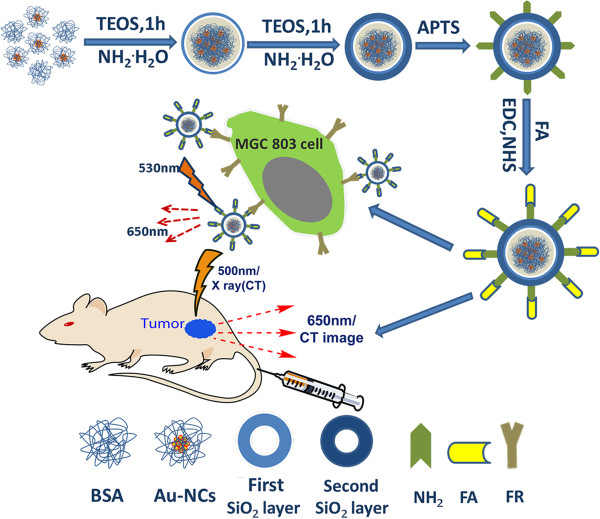
Schematic of experimental course.

In this study, we strategically designed and prepared folic acid conjugated AuNCs@SiO_2_ nanoprobes (AuNCs@SiO_2_-FA), the schematic is shown in Figure 1, selected folic acids and gastric cancer MGC803 cells as research targets, and investigated the feasibility of targeted dual mode fluorescent imaging and CT imaging of in vivo gastric cancer with the aim of laying foundation for further clinical application in near future.

## Results and discussion

### Synthesis and characterization of gold nanoclusters and AuNCs@SiO_2_

AuNCs was synthesized according to a previous method [34]. Exposure of the AuNCs solution to UV (365 nm) lead to an intense red fluorescence signals (Additional file 1: Figure S1, A). Figure 1 shows the TEM images of BSA stabilized AuNCs, the average diameter is 2.42 nm. The inserted magnification picture shows the lattice of Au metal. X-ray photoelectron spectroscopy (Additional file 1: Figure S2) was used to observe the oxidation state of gold. The detected binding energies values were Au4f_7/2_ ~83.9 eV and Au4f_5/2_ ~87.6 eV, respectively [35]. It is valuable that the binding energy of Au4f_7/2_ and Au4f_5/2_ located at the region between the Au(0) binding energy (84 eV) of a metallic gold film and the Au(I) binding energy(86 eV) of gold thiolate, highly suggesting both of Au(0) and Au(I) existed in the BSA-stabilized clusters[30-32]. The energy-dispersive X-ray (EDX) analysis (Additional file 1: Figure S3) showed that the chemical composition of AuNCs was with a presence of Au 6.91%.

**Figure 2:**
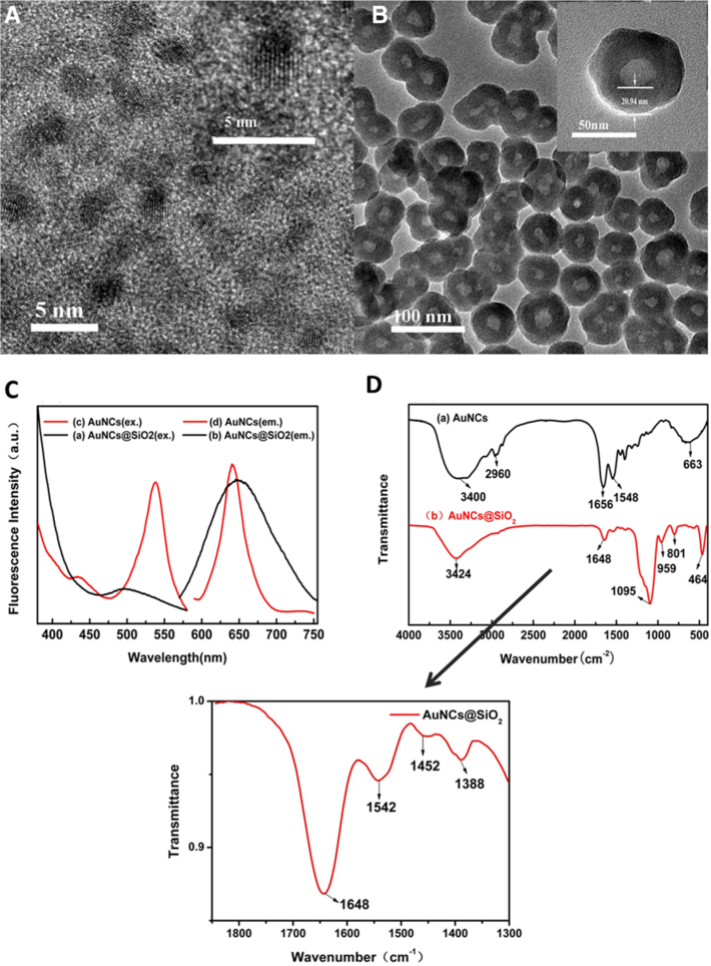
**Experimental procedure of AuNCs@SiO_2_-FA nanoprobes for dual-model imaging.** TEM image of AuNCs (**A**) and AuNCs@SiO_2_ (**B**); (**C**) Excitation and emission spectra of gold nanoclusters (black curve) and AuNCs@SiO_2_ (red curve); (**D**) FTIR spectra of Au-NCs and AuNCs@SiO_2_ samples (powder obtained by freeze-drying).

AuNCs@SiO_2_ was prepared based on a modified Stöber method [36]. Using silica as a matrix shell has a lot of advantages including: it is size-tunable by adjusting the dosage of TEOS and owns good biocompatibility, and it is also easy to make surface modification with different functional groups for further biomedical application. A two-step method was chosen to realize the silica coated Au nanoparticles. The silanization process was carried out by hydrolysis of TEOS with ammonia catalyst. The second step is crucial to form a stable and better distribution matrix for AuNCs, which provide a shielding from the solvent interaction and protect clusters from diffusion outside the silica. The size measurement was performed by TEM images (Figure 1) and Dynamic Light Scattering (Additional file 1: Figure S1, C). It is clear that all silica capped AuNCs@SiO_2_ are approximately spherical in shape with good monodispersity with the average silica-shell thickness of ~20 nm, and total diameter of ~58 nm. The DLS measurement shows the size of AuNCs@SiO_2_ nanoparticles were ~68 nm in diameter, which matched with the TEM result. TEM pictures (Figure 2B, inset) clearly showed that the presence of protein embedded as a core with a thickness ~20 nm, which was due to several gold nanoclusters included by silica shell. The gold core of gold nanoclusters was not easily observed by TEM because they are capped by thick protein-silica matrix and the total content of gold atom is very low [32]. In order to prove that gold nanoclusters were coated in silica shell, an inductively coupled plasma mass spectrometry (ICP-MS) analysis confirmed the presence of AuNCs doped in the silica particles with a presence of 0.0596% Au. To obtain optimal monodisperse spherical nanoparticles, we tested the effects of different dose of TEOS. TEM images of AuNCs@SiO_2_ (B) with adding different doses of TEOS (100 μl + 100 μl, 150 μl + 150 μl, 200 μl + 200 μl) were shown in Additional file 1: Figure S4.

The fluorescence spectra of AuNCs and AuNCs@SiO_2_ are shown in Figure 2C. The left red curve is the excitation spectra of AuNCs, the right red curve is the emission spectra of AuNCs, the left and right black curves are the excitation and emission spectra curves of AuNCs@SiO_2_, respectively. A blue shift was observed in the fluorescence emission spectra, from 665 nm to 645 nm. The blue shift of the emission peak is similar to previous report [32]. We also notice that the Stocks shift (>110 nm) is larger than the conventional organic dyes. The UV–vis absorption spectra (Additional file 1: Figure S2, A) of AuNCs show an obvious peak in the wavelength region 250–350 nm, which correlates with the classic absorbance of aromatic amino acid of BSA. After silica coating, the absorbance peak of 278 nm is disappeared which may be due to the clusters’ surface silica deposition. Both of AuNCs and AuNCs@SiO_2_ show an intense red fluorescence under a UV–vis (365 nm) lamp irradiation (Additional file 1: Figure S1, A, B).

FTIR spectra are used to compare the uncoated AuNCs with AuNCs@SiO_2_ nanoparticles shown in Figure 2D, exhibiting the characteristic vibration peaks of the protein BSA. The characteristic amide I band seen in 1656 cm^-1^ is considered to be associated with the protein secondary structure containing a high portion of ą-helix. The band appearing in 1548 cm^-1^ correspond to strong primary amine scissoring, whereas the band centre at 3400 cm^-1^ can be attributed to primary amines. Peaks located at 2960 cm^-1^ correspond to C-H vibration and a broad band at 663 cm^-1^ can be attributed to –NH_2_ and –NH wagging [31-33] teristic bands of Si-O-Si asymmetric stretch. In addition, a broad band centered at 3424 cm^-1^ can be attributed to the OH stretch originating from both silanol and the absorbed water. Peaks at 959 cm^-1^, 801 cm^-1^, 464 cm^-1^ present show the presence of silanol Si-OH stretch, Si-O-Si symmetric stretch, and Si-O-Si bend, respectively [29-33]. During the coating of silica, the characteristic peaks of AuNCs are covered by the strong peaks of Silica. Even so, we still detected the presence of AuNCs at a low concentration on the AuNCs@SiO_2_ spectra, the results was consistent with a previous report [29]. As shown in inset (Figure 2D, inset), those peaks located at 1648 cm^-1^, 1542 cm^-1^, 1452 cm^-1^, 1388 cm^-1^ are associated with the vibration modes of amide I and amide II band and the stretching of the carboxyl groups.

### Synthesis and characterization of AuNCs@SiO_2_-FA

**Figure 3:**
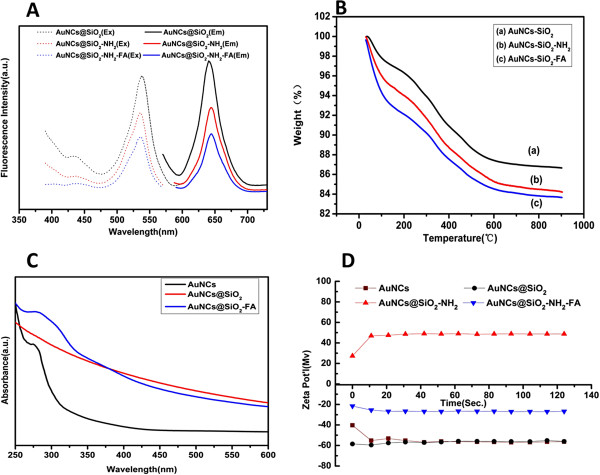
Fluorescence spectrum (A), TGA curve (B), of AuNCs@SiO_2_, AuNCs@SiO_2_-HN_2_ and AuNCs@SiO_2_-FA; (C) UV–vis spectra of AuNCs, AuNCs@SiO_2_ and AuNCs@SiO_2_-FA; (D) Zeta potential of AuNCs, AuNCs@SiO_2_, AuNCs@SiO_2_-HN_2_ and AuNCs@SiO_2_-FA.

A classic silane coupling agent APTS was used to functionalize AuNCs@SiO_2_. A traditional EDC-NHS method was used to activate free carboxyl groups of FA, and then the activated groups combined with the amine group of AuNCS@SiO_2_-NH_2_, resulting in formation of AuNCs@SiO_2_-FA. Figure 3A showed the fluorescence spectra of AuNCs@SiO_2_ (20 mg/ml), AuNCs@SiO_2_-NH_2_(14 mg/ml) and AuNCs@SiO_2_-FA(10 mg/ml). Compared with AuNCs@SiO_2_, the excitation and emission peak of AuNCs@SiO_2_-FA exhibited a little shift. Thermal gravimetric analysis (TGA) was measured between 30°C to 900°C. The weight loss of AuNCs@SiO_2_, AuNCS@SiO_2_-NH_2_ and AuNCs@SiO_2_-FA are calculated at 900°C, which show the average weight loss of 14.3, 15.8 and 16.3 wt%, respectively (Figure 3B). The weight loss of AuNCs@SiO_2_-FA mainly contributes to the disappearance of FA and the part of APTS [34]. Figure 3C showed the absorbance spectra of AuNCs, AuNCs@SiO_2_ and AuNCs@SiO_2_-FA, the BSA protein peak in the near 280 nm disappeared after surface functionalization. Nevertheless, during the region of 270 nm to 310 nm, a broad band comes up after being FA conjugation, which is accorded with the characteristic absorbance of FA, indicating FA was successfully conjugated with AuNCs@SiO_2_. Zeta potential are recorded at pH7.0 shown in Figure 3D, confirming the successful conjugation of NH_2_ and FA. Before functionalized with APTS, the zeta potential of AuNCs@SiO_2_ is −56.02 mV, showing the existence of a lot of OH groups. While, amine functionalized AuNCs@SiO_2_-NH_2_ present a positive zeta potential of +48.78 mV. As an evidence for AuNCs@SiO_2_ successfully conjugated with FA, the zeta potential of AuNCs@SiO_2_-FA showed a negative value of −26.74 mV as shown in Figure 3D (blue curve), which is mainly contributed by the carboxyl groups of FA.

### Cytotoxicity assessment of AuNCs@SiO_2_-FA

**Figure 4:**
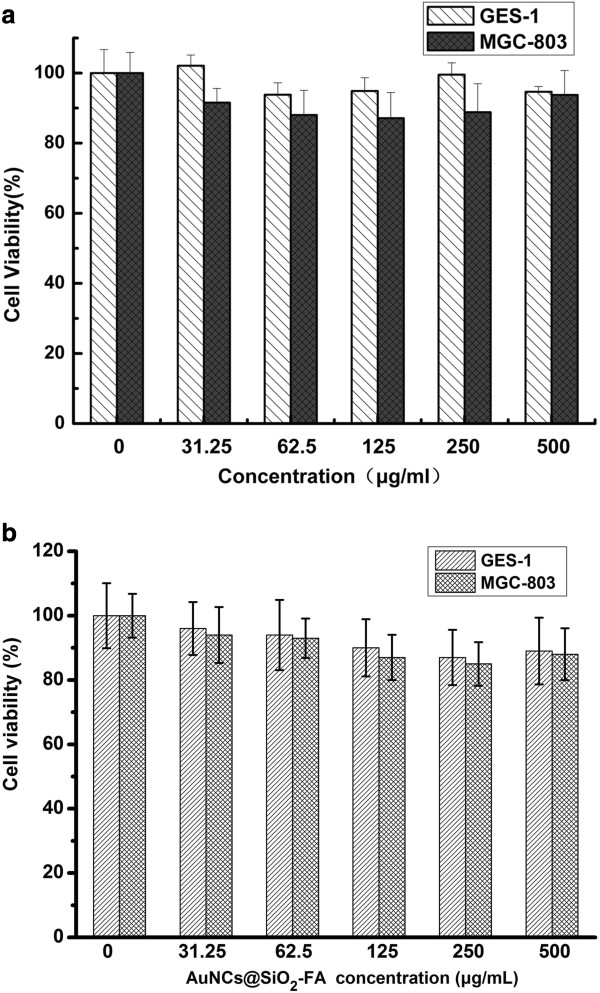
Cell viabilities of MGC-803 cells and GES-1 cells incubated with AuNCs@SiO_2_ (A) and AuNCs@SiO_2_-FA (B) with different concentrations (31.25, 62.5, 125, 250, 500 μg/ml) for 16 h.

It is crucial to evaluate the toxicity profile of nanoprobes for biomedical applications. MGC-803 cell line is from human gastric mucosal cancer cell, while GES-1 cell is from the normal gastric mucous cell. We used MTT method to investigate the cytotoxicity of AuNCs@SiO_2_ and AuNCs@SiO_2_-FA to both MGC-803 cells and GES-1 cells. The cells were treated with different concentrations of AuNCs@SiO_2_ and AuNCs@SiO_2_-FA for 16 hours at 37°C under 5% CO_2_, respectively. The concentrations (31.25-500 μg/ml) were kept consistent for both MGC-803 cells and GES-1 cells. As shown in Figure 4A and 4B, all the cell viabilities were more than ~80%, the AuNCs@SiO_2_ and AuNCs@SiO_2_-FA did not show a marked toxicity to the two kinds of cells, even up to the relatively higher concentrations of 500 μg/ml. Although AuNCs@SiO_2_-FA nanoprobes could enter into MGC803 cells, the nanoprobes did not show stronger toxicity to MGC803 cells than AuNCs@SiO_2_, no statistical difference existed between AuNCs@SiO_2_-FA and AuNCs@SiO_2_ (*P* >0.5).

### Fluorescent imaging of MGC-803 Cells by confocal microscope

Folate receptor (FR), is a glycosylphosphatidyinositol-linked high-affinity membrane protein, commonly expressed on the surface of many human cancer cells. Folic acid (FA), is a water-soluble vitamin (B_9_), which displays high affinity for the folate receptor that captures its ligand from the extracellular milieu and transports them into the cytoplasm via a non-destructive, recycling endosomal pathway. Therefore, we prepared red-emitting fluorescence AuNCs@SiO_2_-FA nanoprobes with the aim of investigating the feasibility of as-prepared nanoprobes to target MGC803 cells based on FA-FR-mediated endocytosis. In our previous work [36], we used flow cytometer to analyze the FR expression on the surface of MGC803 cells and GES-1 cells, results showed that FR were over-expressed on the surface of MGC803 cells, no expression on the surface of GES-1 cells.

**Figure 5:**
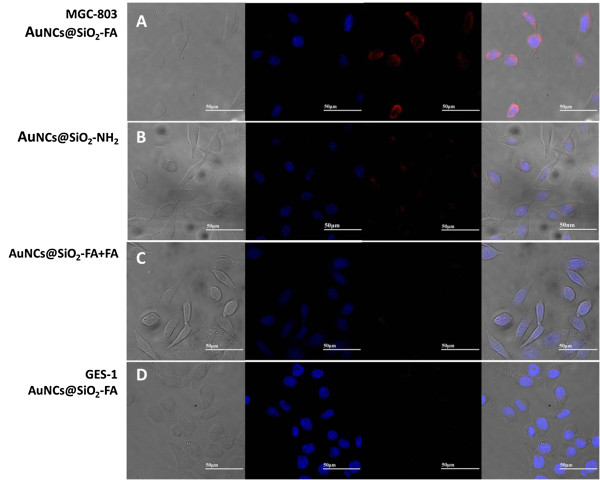
Confocal fluorescence imaging of MGC-803 cells incubated with AuNCs@SiO_2_-FA (a), AuNCs@SiO_2_-NH_2_(b), AuNCs@SiO_2_-FA with excess FA (c), and GES-1 at 500 μg/ml incubated with AuNCs@SiO_2_-FA (d) at 500 μg/ml.

To investigate AuNCs@SiO_2_-FA nanoprobes’ targeted ability, a total of four sets of experiments were designed. One experimental group was set as to exploit the positive absorbance, two negative group trials and a competitive group trial were set as controls. First of all, both MGC-803 cells (Figure 5A) and GES-1 cells (Figure 5D) were treated with AuNCs@SiO_2_-FA, which were set as the targeted specificity group and negative control group, respectively. Then, a non-specific group was tested by treated the same batch of MGC-803 cells with AuNCs@SiO_2_-NH_2_ (Figure 5B), which was set as an another negative control. In another set of experiment, a competed experiment was used as a further proof to illuminate the FR-mediated target delivery by treating the same batch of MGC-803 cells with both AuNCs@SiO_2_-FA and excess free FA as shown in Figure 5C. All the results were obtained at the concentration of 500 μg/ml nanoprobes under incubation at 37°C for 1.5 hour.

As shown in Figure 5, from left to right, those images such as the bright-field, dark-field blue fluorescence images of nucleus, red fluorescence images and the overlay images, were presented respectively. First of all, Figure 5A showed the result of AuNCs@SiO_2_-FA targeted experiment group, which clearly showed that some red fluorescence surrounded the blue nucleus in the MGC803 cells, indicating that some AuNCs@SiO_2_-FA nanoprobes crossed the cell membrane and entered into the cytoplasm, and some accumulated near the perinuclear region. Figure 5B showed the result of MGC803 cells incubated with AuNCs@SiO_2_-NH_2_, showing very low red fluorescence in the red channel, which may be nonspecific uptake in nature. Figure 5C showed the result of competitive test, which was as expected, rare low red fluorescence intensity could be seen in MGC803 cells. Figure 5D showed the result of GES-1 cells incubated with AuNCs@SiO_2_-FA nanoprobes, no obvious red fluorescence signal could be seen in the GES-1 cells. Therefore, prepared AuNCs@SiO_2_-FA nanoprobes can specifically target MGC803 cells.

### AuNCs@SiO_2−_FA nanoprobes for fluorescent imaging of in vivo gastric cancer cells

**Figure 6:**
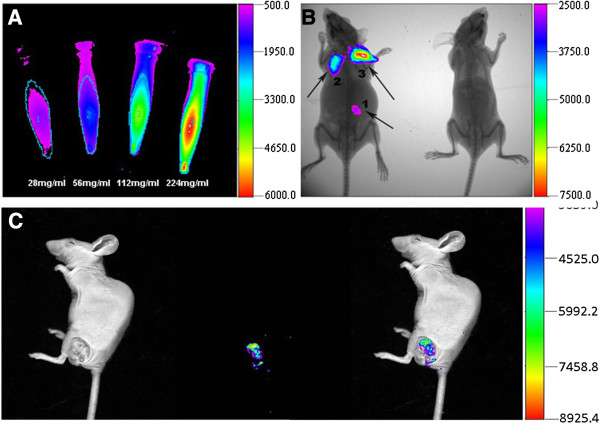
**AuNCs@SiO_2_-FA nanoprobes for fluorescence imaging.** (**A**) In vitro fluorescence image of AuNCs@SiO_2_–FA in 0.01 M PBS with different concentrations; (**B**) In vivo fluorescence image of 50ul AuNCs@SiO_2_-FA injected subcutaneously at three different dose (area-1: 56 mg/ml; area-2: 112 mg/ml; area-3: 226 mg/ml) into the left mice. The left mice without injected was selected as control; (**C**) Fluorescence image of tumor tissues with tail vein injection at the concentration of 287 mg/ml at 6 h post-injection.

**Figure 7:**
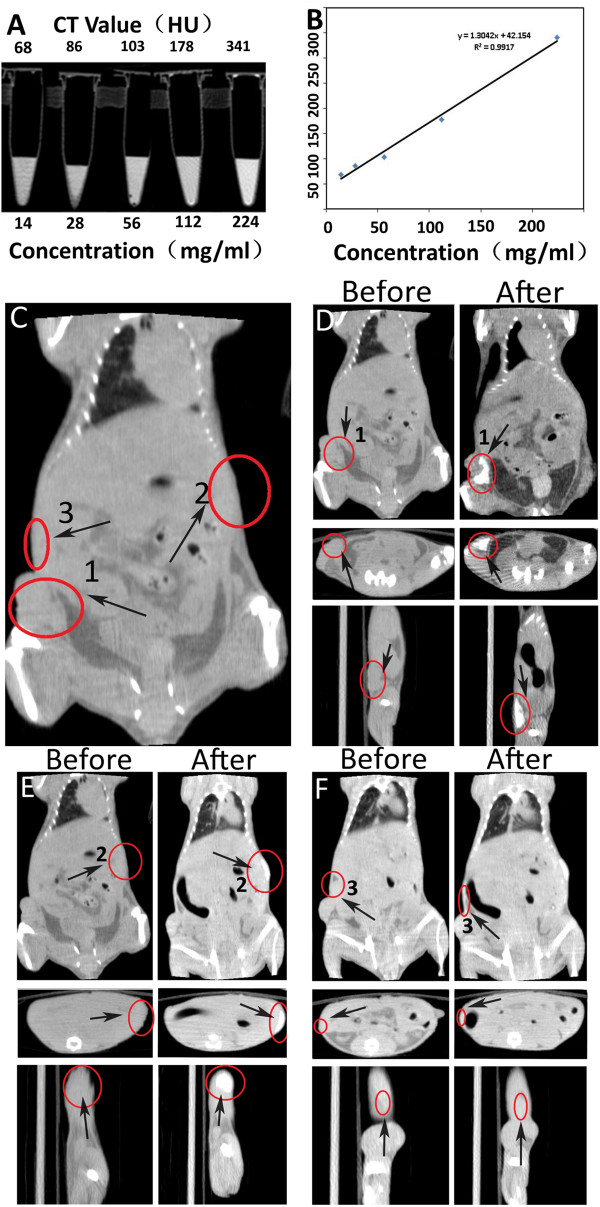
**AuNCs@SiO_2_-FA nanoprobes for CT imaging.** (**A**) In vitro CT images of AuNCs@SiO_2_-FA in 0.01 M PBS. The concentration (mg/mL) in each sample is provided at the bottom of the respective images; (**B**) CT attenuation (HU) plot of AuNCs@SiO_2_-FA at various concentrations in the range from 14 to 226 mg/ml. In vivo CT images of nude models before and after tail vein and subcutaneous local injection: (**C**) showed the two different sites and the control site, named area-1(tumor site), area-2(back site), area-3 (soft tissue), respectively; (**D**) and (**E**) showed the CT images before and after tail vein and subcutaneous injection of area-1 and area-2 respectively. (**F**) area-3 soft tissue was selected as a control to area-1 and area-2.

To investigate the feasibility of AuNCs@SiO_2_-FA nanoprobes targeted in vivo gastric cancer cells in nude mice models, fluorescent signal of AuNCs@SiO_2−_FA nanoprobes was tested in vitro at first. Four different concentrations of AuNCs@SiO_2−_FA in PBS were scanned with a Carestream FX PRO Image System (Carestream Health, USA). As shown in Figure 6A, as the concentration of AuNCs@SiO_2_-FA nanoprobes increased, the optical signal intensity also increased. The result highly indicated that AuNCs@SiO_2−_FA nanoprobes could be used for in vivo imaging. Then, the AuNCs@SiO_2−_FA nanoprobes were subcutaneously injected into the back of nude mice at three dose (56 mg/ml, 112 mg/ml, 224 mg/ml), the injected sites were named as area-1, area-2 and area-3, respectively, as shown in Figure 6B. A nude mouse without injection was selected as the control. These mice were imaged immediately after injection by using a Carestream FX PRO Image System (Carestream Health, USA). As shown in Figure 7B, all three injection sites exhibited bright and strong fluorescent signals. The fluorescence intensity was enhanced with the increase of concentration of AuNCs@SiO_2_-FA.

In order to investigate the feasibility of AuNCs@ SiO_2_-FA nanoprobes’ targeted in vivo gastric cancer, the as-prepared nanoprobes were injected into nude mice models via tail vein. Figure 6C showed clearly the strong fluorescent imaging at the tumor location at 6 h post-injection, which highly suggest that prepared AuNCs@SiO_2_-FA nanoprobes can target in vivo gastric cancer cells, and can be used as contrast reagent for optical imaging in vivo.

### AuNCs@SiO_2_-FA nanoprobes for CT imaging of in vivo gastric cancer

When AuNCs@SiO_2_–FA nanoprobes were confirmed to be effectiveness for in vivo fluorescence imaging, we continued to investigate the feasibility of AuNCs@SiO_2_-FA nanoprobes for CT imaging of in vivo gastric cancer. As one of the most important molecular imaging diagnostic technologies, CT imaging provides better spatial and density resolution than other imaging modalities [36,37]. Conventional CT contrast agents (CAs) are based on iodinate small molecules because iodine has a highest X-ray absorption coefficient among nonmetal atoms [22-24], but exists several inherent shortcomings [12,23-25]. Iodinate molecules do not allow long-time imaging due to the rapid clearance by the kidney, and also may cause renal toxicity. Recently, some nanoparticles contains gold atom have been actively used for CT imaging, and also gain satisfactory results [24-26,37], which is because gold has a higher X-ray absorption coefficient (5.16 and 1.94 cm^2^/g, respectively, at 100 keV) than iodine agents. Therefore, we investigated the possibility of use of AuNCs@SiO_2_–FA nanoprobes as a CT imaging contrast agent in vivo.

At first, we tested the in vitro CT images of AuNCs@SiO_2_–FA nanoprobes. As shown in Figure 7A, as the mass concentration of AuNCs@SiO_2_–FA nanoprobes increased, the CT signal intensity also continuously increased. To investigate the CT contrast effects, the attenuation values (HU) of different concentrations of nanoprobes were performed by a Micro-CT equipment. As shown in Figure 7B, HU as a function of the concentration of AuNCs@SiO_2_–FA nanoprobes exhibited a well-correlated linear relationship (R^2^ = 0.9917), and was described by the following typical equation: Y = 1.3042X + 42.154. For in vivo CT imaging, a relative higher concentration of AuNCs@SiO_2_–FA nanoprobes were respectively injected into nude models loaded with gastric cancer via tail vein, and were injected into the back of mice, as shown in Figure 7C named as area-1(tumor site) and area-2 (back site) (marked by red circle) respectively, and area-3 without injection was used as a control. Pre-injection CT images were used as references, and were shown in Figure 7D (before) and Figure 7D (after). Pre-injection HU values of two sites were estimated to be 129.16 and 132.58, post-injection HU values of two sites were respectively 383.32 and 426.13, the after-to-before signal ratios were respectively 3.83 and 4.26, presented distinguished CT signals (Figure 7D, Figure 7E, after). Two distinct concentrations of nanoprobes injection into the nude mouse also was tested as shown in Figure S5. Figure 7F showed the images of the mice soft tissue area-3 without injection, the HU values of pre-injection and post-injection were respectively 90.21 and 101.36, the after-to-before ratio was 1.013, CT signals did not change too much, indirectly showing that as-prepared nanoprobes own good tumor site-targeted ability. Therefore, our results fully show that prepared AuNCs@SiO_2_–FA nanoprobes might be a promising CT imaging contrast agent for in vivo gastric cancer.

## Conclusion

In summary, a folic acid conjugated silica coated AuNCs nanoprobe was successfully designed and developed, which have a uniform sphere shape and size distribution, good water solubility, fluorescent photostability and biocompatibility. The prepared AuNCs@SiO_2_-FA nanoprobes show effectively targeted ability to the FR(+) MGC-803 cells, while few uptake existed in FR(−) GES-1 cells. The prepared nanoprobes also exhibit excellent red emitting fluorescence optical property and X-ray absorbance for optical and CT dual-modality imaging of gastric cancer. The prepared AuNCs@SiO_2_-FA nanoprobes own great potential in applications such as targeted fluorescent and CT dual mode imaging of in vivo early gastric cancer in near future.

## Methods

### Materials

Deionized water (Millipore Milli-Q grade) was used to prepare all aqueous solutions. Chloroauric acid (HAuCl_4_), 3-aminopropyltrimethoxysilane (APTS), 1-ethyl-3-(3-dimethyl aminopropyl) carbodiimide (EDC), N-hydroxysuccinimide (NHS), folic acid (FA), tetraethoxysilane(TEOS, 99.9%), bovine serum albumin(BSA) and ascorbic acid were purchased from Shanghai Aladdin-Reagent Co., Ltd. Anhydrous ethanol, NaOH and ammonia (NH_3_.H_2_O) were obtained from Sinopharm Chemical Co. (China). All chemicals were analytical grade and used directly without any purification.

### Synthesis and characterization of gold nanoclusters (AuNCs)

Gold nanoclusters were prepared based on a reference [34]. Briefly, 10 mL aqueous solution of HAuCl_4_ was added into 10 ml 50 mg/ml bovine serum albumin under vigorous stirring at 37°C. The solution was mixed for two minutes. Then, 250ul ascorbic acid was added dropwisely. Five minutes later, 1 ml of 1 M NaOH solution was introduced to adjust the PH, and the mixture solution was incubated at 37°C for another 9 hours. The color of the solution transformed into light brown and finally changed into deep brown. The resultant AuNCs were kept in 4°C for usage. Fluorescence spectra of gold nanoclusters were performed on an F-4600 Fluorescence spectrophotometer (Hitachi, Japan). UV–vis extinction spectra was scanned by a Varian Cary50 UV-via spectrophotometer (Varian, USA) in transmission mode. The surface atom composition of gold nanoclusters was measured by an AXIS LUTRADLD X-ray photoelectron spectroscopy (XPS) (Kratos, Japan). Thermal gravimetric analysis (TGA) was detected by using a Pyris 1 TGA (Pekin Elmer, USA). The temperature was increased from 20 to 900°C at a rate of 10°C/min. Transmission electron microscopy images were carried out by using a JOEL Model JEM-2010 instrument (JOEL Ltd., Germany). Fourier transform infrared (FTIR) spectra were measured by using a Nicolet 6700 (Thermo Fisher, USA). Zeta potentials were detected by the NICOMP 380ZLS zeta potential/particle size. Dynamic light scattering (DLS) was performed on NANO/ZEN 1600 system (Malvern Instruments Ltd., U.K.).

### Preparation and characterization of silica-coated gold nanoclusters (AuNCs@SiO_2_)

In a typical experiment, silica was used to obtain a core-shell structure fluorescence nanoparticles via a modified Stöber method [33]. Firstly, 200 μl Au-NCs were diluted in 20 ml alcoholic solution containing 800 μl ammonia by sonication for 5 minutes. Next, 200 μl tetraethylorthosilicate (TEOS) was added into the mixture under stirring for 1 hour. Lastly, another 200 μl TEOS were added and followed with vigorous stirring for 24 hours. The fluorescent nanoparticles were collected by centrifuged, washed for three times with absolute ethanol and deionized water. The purified sample named as AuNCs@SiO_2_ was diluted in the deionized water at 20 mg/ml and characterized by UV–vis absorption spectra.

### NH_2_-functionalization and FA-conjugation of AuNCs@SiO_2_

The AuNCs@SiO_2_ solution was used to conjugate with folic acids. In a typical procedure, 2 ml of the obtained solution was added into 40 ml ethanol, then 7 ml of APTS was added into the solution, the mixture was continued to react for 4 hours under gently stirring at 35°C. Finally, the product was separated by centrifugation, and washed for three times with deionized water and absolute ethanol, the final product was AuNCs@SiO_2_-NH_2._

AuNCs@SiO_2_-NH_2_ was conjugated with folic acid (FA) according to the following procedure. Briefly, FA was diluted with dimethyl sulfoxide (DMSO) under sonication to ensure complete dispersion. Then, EDC and NHS were added as covalent coupling reagents and the solution was kept for 1 hour at room temperature under stirring. Next, the obtained AuNCs@SiO_2_-NH_2_ solution was added into the activated FA solution, the mixture was kept stirring for another 3 hours. The FA-conjugated AuNCs@SiO_2_ nanoparticles were collected by centrifugation, and washed three times with PBS, and then were kept at 4°C for usage.

### Cytotoxicity evaluation of AuNCs@SiO_2_ and AuNCs@SiO2-FA

MGC-803 cells and GES-1 cells were cultured at 37°C under 5% CO_2_, in RPMI 1640 supplemented with 10% NBS and 1% penicillin/sterptromycin. The cytotoxicity of AuNCs@SiO_2_ and AuNCs@SiO_2_-FA to MGC-803 cells and GES-1 cells were measured by a 3-(4,5- dimethylthiazol-2-yl)-2, 5-diphenyltetrazolium bromide (MTT) assay. MGC-803 cells and GES-1 cells were seeded into a 96-well cell-culture plate at 10^5^ cells per well in a 150 μl volume and incubated for 24 hours at 37°C under 5% CO_2._ After being washed with PBS, the cell media were replaced by adding 150 μl per well RPMI solutions of AuNCs@SiO_2_–FA with various concentrations (31.25, 62.5, 125, 250, 500 μg/ml). Positive controls were replaced by fresh 10% NBS-containing media. Five wells were set up as positive controls for each sample concentration. After being incubated for 16 hours under the same condition, 20 μl of MTT stock solution (5 mg ml^-1^) was added into each well and kept for 4 hours. The culture media were removed and the performed crystals were dissolved in 100 μl DMSO. The optical absorbance was determined using a microplate reader at 570 nm.

### Fluorescence imaging of gastric cancer cells by confocal microscope

Gastric cancer MGC-803 cells incubated with AuNCs@SiO_2_-FA were imaged with a Leica TCS SP5-II confocal fluorescence microscope, equipped with a color CCD camera (Leica, USA). DAPI emission was recorded between 440 and 470 nm;AuNCs emission images were obtained using a 590 nm long-pass filter. 500 μg/ml AuNCs@SiO_2_-FA solution were added into cultured MGC-803 and GES-1 cells, and incubated for 1.5 h at 37°C under 5% CO_2_%. After being washed with PBS (0.01 M, pH 7.4) for three times, the cell nuclei were stained with DAPI (4′-6-diamidino-2-phenylindole) for 5 min. Subsequently, the stained cells were washed with PBS for three times, and fixed with 2% paraformaldehyde for 15 min, and then mounted with the mounting medium. As negative control, GES-1 cells were cultured at the similar condition in RPMI 1640 medium with 10% NBS serum. Further, AuNCs@SiO_2_-NH_2_ and AuNCs@SiO_2_-FA with excess FA were incubated with MGC-803 cells under the same condition were set up as negative controls.

### Establishment of animal models

All animal studies complied with current ethical considerations with the approval (SYXK-2007-0025) of the Institutional Animal Care and Use Committee of the Shanghai JiaoTong University. SCID mice (male, 20–22 g, 5 weeks old) were obtained from the Shanghai LAC Laboratory Animal Co. Ltd., Chinese Academy of Sciences (SCXK2007-0005) and housed in SPF grade animal center. Those 5-week-old male nude mice were subcutaneously injected in the right side of their back with 1×10^6^MGC803 ells/mouse. When the tumor nodules reached a volume of about 5.0 mm^3^ after approximately 3 weeks post-injection, the tumors were confirmed by gross specimen.

### AuNCs@SiO-FA nanoprobes for fluorescent imaging of in vivo gastric cancer

Subcutaneous experiments were performed to demonstrate the effectiveness of the AuNCs@SiO-FA nanoprobes for in vivo optical imaging. Firstly, 50 μl of AuNCs@SiO_2_ –FA dissolved in 0.01 M PBS solution was substaneously injected into the back of the nude mices with three different concentrations (224 mg/ml, 112 mg/ml and 56 mg/ml). A nude mouse without injection of AuNCs was selected as the control. Then 50 μl of AuNCs@SiO_2_ –FA solution was injected into nude mice via tail vein. In vivo imaging was performed at 6 h post-injection by using a Carestream FX PRO Image System (Carestream Health, USA) with 600 nm emission filter and 530 nm excitation filter. All counts were acquired on the same workstation using the software supplied by manufacture with the same parameters.

### AuNCs@SiO-FA nanoprobes for CT imaging of in vivo gastric cancer

CT scanning was performed both before and after tail vein and subcutaneously injection of FA-AuNCs@SiO_2_ at the concentration of 224 mg/ml, respectively. The attenuation value (HU) of pre-injection scanning was considered as a reference. The CT imaging of nude mice models were performed using a Siemens Inveon Multimodality PET/CT system (Siemens Medical Solutions, Inc., Knoxville, Tennessee). The CT imaging parameters were as follows: effective pixel size, 105.61 μm; 80 KVp, 500 μA; field of view, 47.90 mm × 98.00 mm; rotation steps, 180; binning, 4; exposure time, 150 ms/rotation. Images were acquired and analyzed using Inveon Research Workplace software (Siemens, Berlin, Germany).

### Statistical analysis

Each experiment was repeated three times in duplicate. The results were presented as mean ± SD. Statistical differences were evaluated using the t-test and considered significance at P < 0.05.

## Competing interests

The authors declare that they have no competing interests.

## Authors’ contributions

ZZ and CZ carried out synthesis and characterization of gold NCs, QQ and JM finished MTT analysis, XZ, LP, GG finished the fluorescent imaging, ZZ, PH and SF carried out the CT imaging, XZ and HS prepared nude mice model loaded with gastric cancer, DC and NJ participated in the design of the study and performed the statistical analysis, and revised the manuscript. All authors read and approved the final manuscript.

## Supplementary Material

Additional file 1 **Folic Acid-conjugated Silica capped Gold Nanoclusters for Targeted Fluorescence/X-ray Computed Tomography Imaging. Figure S1.** Pictures of AuNCs (A) and AuNCs@SiO_2_ (B) show intense red fluorescence under a UV-vis (365 nm). (C) Dynamic Light Scattering of AuNCs (blue curve) and AuNCs@SiO_2_ (red curve). **Figure S2.** (A) Us-Vis spectrum of AuNCs. (B) X-ray photoelectron spectroscopy of AuNCs. lamp irradiation. The XPS binding energies values show Au4f_7/2_ ~83.9 eV and Au4f_5/2_ ~87.6eV, respectively. It is valuable that the banding energy of Au4f_7/2_ and Au4f_5/2_ locate at the region between the Au(0) binding energy (84 eV) of a metallic gold film and the Au(I) binding energy (86 eV) of gold thiolate, suggesting the exhibition both of Au(0) and Au(I) in the BSA-stabilized clusters [28,29]. **Figure S3.** Energy Dispersive X-Ray Spectroscopy (EDX) of AuNCs. **Figure S4.** TEM images of AuNCs@SiO_2_ (B) with adding different doses of TEOS (100 μl, 150 μl, 200 μl, from left to right) one time. All experiments were under the same operation and conditions. When TEOS was increased to 200 μl, the obtained nanoparticles show monodisperse spherical nanoparticles. Figure S5. CT images of subcutaneous pre-injection and post-injection of nude models with gastric cancer with AuNCs@SiO2-FA nanoprobes in 0.01 M PBS at the concentration of 226 mg/ml and 56 mg/ml. Click here for file
