# Supplementary material for: Folic acid-conjugated silica capped gold nanoclusters for targeted fluorescence/X-ray computed tomography imaging
Source: J Nanobiotechnology. 2013 May 29;11:17. doi: 10.1186/1477-3155-11-17 (PMC3669628; doi:10.1186/1477-3155-11-17)
Supplement: Additional file 1 — Folic Acid-conjugated Silica capped Gold Nanoclusters for Targeted Fluorescence/X-ray Computed Tomography Imaging. Figure S1. Pictures of AuNCs (A) and AuNCs@SiO2 (B) show intense red fluorescence under a UV-vis (365 nm). (C) Dynamic Light Scattering of AuNCs (blue curve) and AuNCs@SiO2 (red curve). Figure S2. (A) Us-Vis spectrum of AuNCs. (B) X-ray photoelectron spectroscopy of AuNCs. lamp irradiation. The XPS binding energies values show Au4f7/2 ~83.9 eV and Au4f5/2 ~87.6eV, respectively. It is valuable that the banding energy of Au4f7/2 and Au4f5/2 locate at the region between the Au(0) binding energy (84 eV) of a metallic gold film and the Au(I) binding energy (86 eV) of gold thiolate, suggesting the exhibition both of Au(0) and Au(I) in the BSA-stabilized clusters [28,29]. Figure S3. Energy Dispersive X-Ray Spectroscopy (EDX) of AuNCs. Figure S4. TEM images of AuNCs@SiO2 (B) with adding different doses of TEOS (100 μl, 150 μl, 200 μl, from left to right) one time. All experiments were under the same operation and conditions. When TEOS was increased to 200 μl, the obtained nanoparticles show monodisperse spherical nanoparticles. Figure S5. CT images of subcutaneous pre-injection and post-injection of nude models with gastric cancer with AuNCs@SiO2-FA nanoprobes in 0.01 M PBS at the concentration of 226 mg/ml and 56 mg/ml. [file 1477-3155-11-17-S1.docx]

**Supplementary information**

**Folic Acid-conjugated Silica capped Gold Nanoclusters for Targeted Fluorescence/X-ray Computed Tomography Imaging**

Zhijun Zhou^1^, Chunlei Zhang^1^, Qirong Qian^2^, Jiebing Ma^1^, Peng Huang^1^, Xizhang^1^, Liyuan Pan^1^, Guo Gao^1^, Hualin Fu^1^, Shen Fu^3^, Hua Song^1^, Xiao Zhi^1^,Jian Ni^1^, Daxiang Cui^1^*


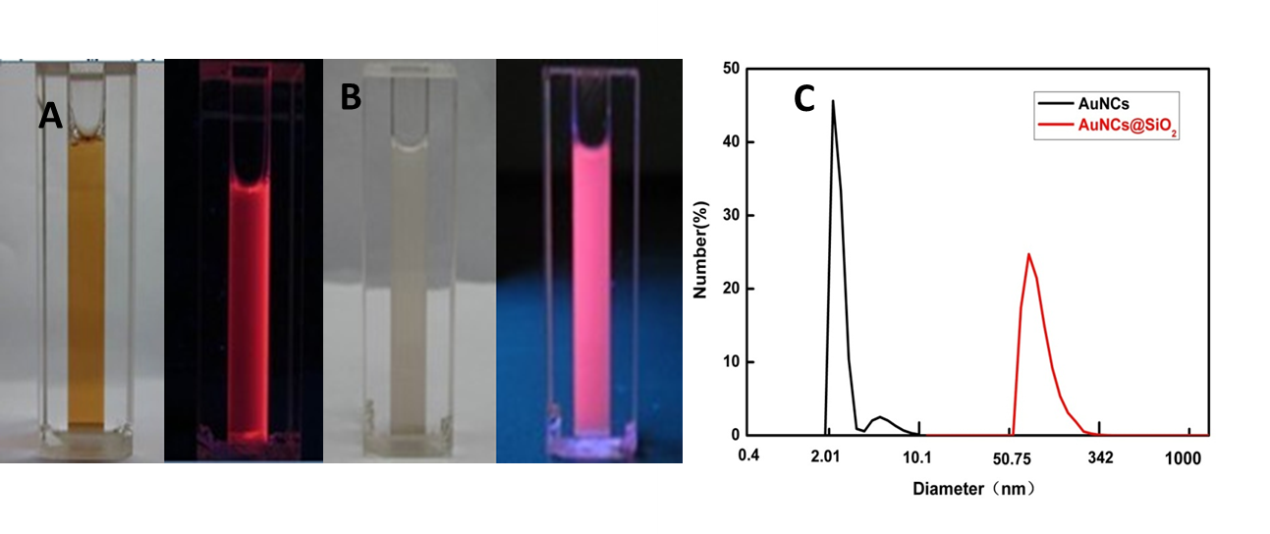


Figure S1. Pictures of AuNCs (A) and AuNCs@SiO_2_ (B) show intense red fluorescence under a UV-vis (365nm). (C) Dynamic Light Scattering of AuNCs (blue curve) and AuNCs@SiO_2_ (red curve).


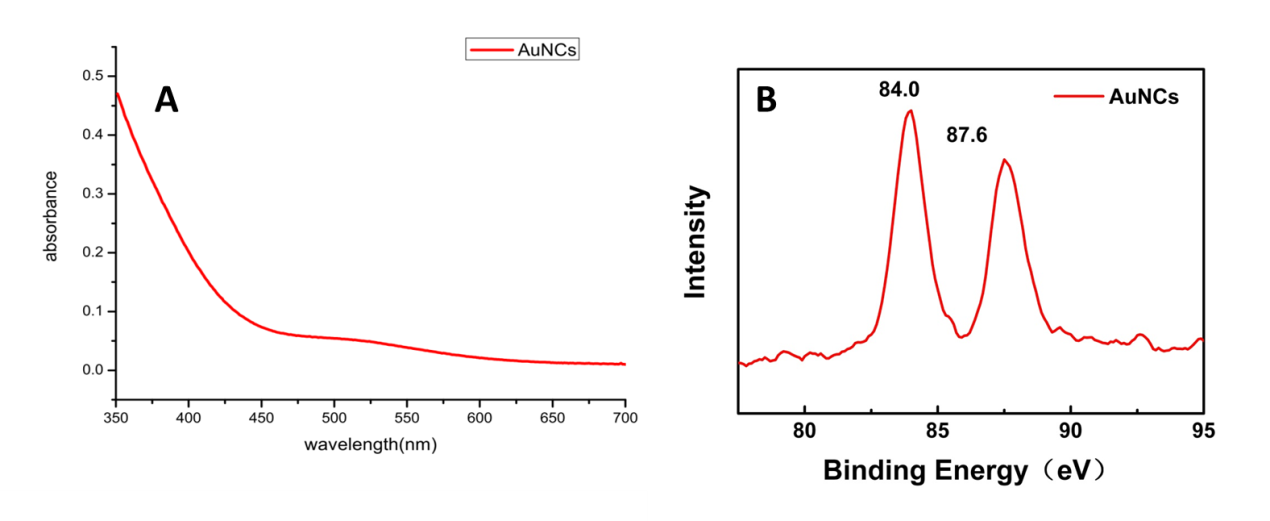


Figure S2. (A) Us-Vis spectrum of AuNCs. (B) X-ray photoelectron spectroscopy of AuNCs. lamp irradiation.

The XPS binding energies values show Au4f_7/2_ ~83.9 eV and Au4f_5/2_ ~87.6eV, respectively. It is valuable that the banding energy of Au4f_7/2_ and Au4f_5/2_ locate at the region between the Au(0) binding energy (84 eV) of a metallic gold film and the Au(I) binding energy(86 eV) of gold thiolate, suggesting the exhibition both of Au(0) and Au(I) in the BSA-stabilized clusters[28-29].


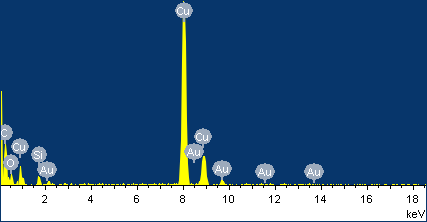


Figure S3. Energy Dispersive X-Ray Spectroscopy (EDX) of AuNCs.


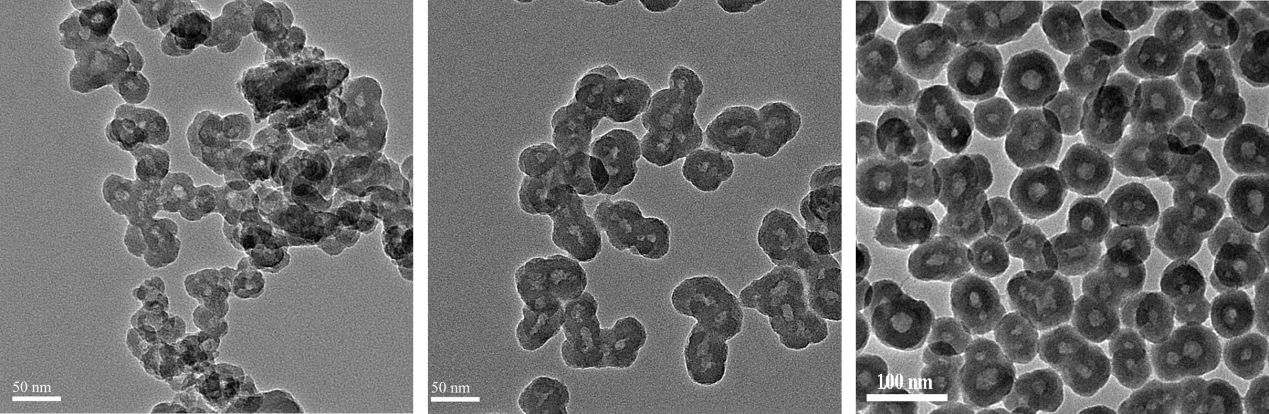


Figure S4. TEM images of AuNCs@SiO_2_ (B) with adding different doses of TEOS (100μl, 150μl, 200μl, from left to right) one time. All experiments were under the same operation and conditions. When TEOS was increased to 200μl, the obtained nanoparticles show monodisperse spherical nanoparticles.


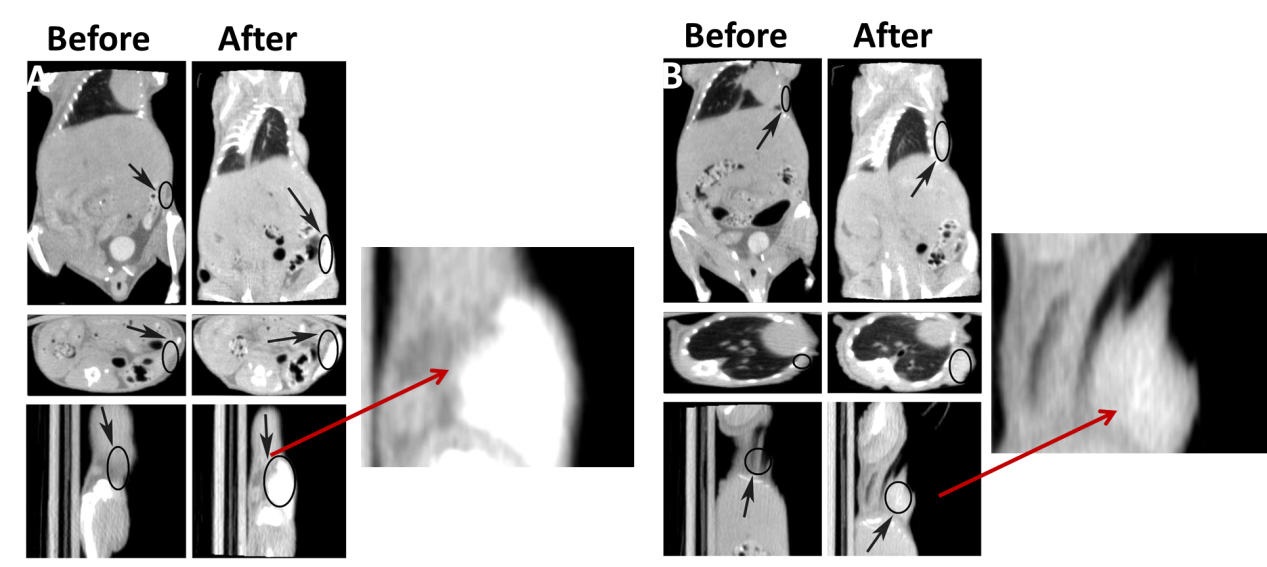
.

Figure S5. CT images of subcutaneous pre-injection and post-injection of nude models with gastric cancer with AuNCs@SiO2-FA nanoprobes in 0.01M PBS at the concentration of 226mg/ml and 56mg/ml.
